# Substrate Oxidation Does Not Influence Middle Distance Running Performance: A Randomized Controlled Crossover Trial

**DOI:** 10.3390/nu17172771

**Published:** 2025-08-27

**Authors:** Alex Buga, Jeffrey D. Buxton, Emma Plank, James D. Minor, Micah T. Sterrett, Christopher A. Brooks, Tanner R. Niemann, Margaret P. Troxel, Anthony Bryarly, Zachary Furry, Clarra Hannon, Jason Muench, Daniel Stone, Dominic P. D’Agostino, Jeff S. Volek, Andrew P. Koutnik, Philip J. Prins

**Affiliations:** 1 Department of Human Sciences, The Ohio State University, Columbus, OH 43210, USA; buga.1@osu.edu (A.B.); volek.1@osu.edu (J.S.V.); 2 Department of Exercise Science, Grove City College, Grove City, PA 16127, USA; buxtonjd@gcc.edu (J.D.B.); plankee20@gcc.edu (E.P.); jd.minor@outlook.com (J.D.M.); mtsthomas98@gmail.com (M.T.S.); cbrooks00100@gmail.com (C.A.B.); niemanntanner17@gmail.com (T.R.N.); troxelmaggie@gmail.com (M.P.T.); bryarlyam21@gcc.edu (A.B.); furryza21@gcc.edu (Z.F.); hannoncg19@gcc.edu (C.H.); muenchjd20@gcc.edu (J.M.); stonedj20@gcc.edu (D.S.); 3 Department of Molecular Pharmacology & Physiology, University of South Florida, Tampa, FL 33620, USA; ddagosti@usf.edu; 4 Human Healthspan, Resilience and Performance, Florida Institute for Human and Machine Cognition, Pensacola, FL 32502, USA; andrewkoutnik@gmail.com

**Keywords:** ketogenic, carbohydrate, time trial, supplementation, energy bar

## Abstract

Objective: Recent work has challenged the notion that preferred substrate oxidation is a key determinant of exercise performance. This investigation tested middle-distance running performance, in the fed state, to control for glycogen and exercise-induced hypoglycemia (EIH) confounders. Methods: In a randomized crossover fashion, all while controlling dietary intake, activity, and body weight, recreational distance runners completed either a 5K (*n* = 15; VO_2max_: 58.3 ± 6.2 mL/kg/min) or a 10K (*n* = 15; VO_2max_: 54.51 ± 5.9 mL/kg/min) middle-distance run after consuming isocaloric low-carbohydrate high-fat (LCHF) and high-carbohydrate low-fat (HCLF) pre-exercise meals. Time trial (TT) performance (sec), carbohydrate/fat substrate oxidation, blood metabolites, heart rate (HR), ratings of perceived exertion (RPE), and subjective fullness and thirst were measured throughout. Results: LCHF pre-exercise nutrition reliably altered substrate oxidation and metabolite profiles compared to HCLF, evidenced by significant increases in fat oxidation (77% higher) and reductions in RER (5% lower), with corresponding shifts in carbohydrate oxidation. Despite distinct preferred substrate oxidation profiles during exercise, the 5 and 10 km TT performances were similar between conditions (*p* = 0.646/*p* = 0.118). RER was significantly lower (*p* = 0.002) after the LCHF condition compared to HCLF. Capillary R-βHB increased modestly after LCHF, while blood glucose increased after HCLF only. The LCHF meal was 35% more filling than the HCLF meal. Preferred substrate oxidation did not significantly modulate middle-distance running performance. Conclusion: This work supports recent findings that substrate oxidation is not a primary determinant of aerobic performance, as previously conceived.

## 1. Introduction

Pre- and intra-race nutritional strategies can positively alter athletic performance. Acute carbohydrate ingestion can shorten time trial (TT) performance by replenishing the glucose pool and mitigating exercise-induced hypoglycemia (EIH) during endurance events [1]—colloquially referred to as preventing “bonking” [2,3]. Athletes perform well following both acute [4] and chronic [5] carbohydrate manipulations prior to competition; however, the vast majority—often as high as 90%—experience gastrointestinal discomfort that limits their performance if they consume carbohydrates during a race [6].

Several logical countermeasures exist to offset intra-race refueling discomfort and preserve performance. The first strategy stipulates pre-loading, whereby athletes consume most calories hours prior to the race to offset digestion issues and saturate intra-muscular glycogen stores in preparation for high-intensity exercise demands [7]. The second strategy, albeit not mutually exclusive to the first, relies on precision-nutrition. This nutritional framework seeks to identify the optimal carbohydrate and lipid type/quantity intake strategies based on the athlete’s habitual diet, tolerance, and exercise demands, including other surrogates such as blood biomarkers, predictive of peak performance [8,9].

Inexorably linked to aerobic events is enhanced lipid oxidation. Athletes may reduce the total carbohydrate load to preferentially oxidize lipids during 45–65% VO_2max_ steady-state exercise, commensurable with a decrease in skeletal muscle glycogen availability [10,11]. Although it has been well-demonstrated that carbohydrates supplant the energy requirements at exercise intensities > 65% VO_2max_ (i.e., cross-over concept), emerging research suggests that macronutrient manipulations can plausibly shift the cross-over point to higher VO_2max_ thresholds than previously conceived [12], an effect that can predictably preserve greater rates of fat oxidation during endurance exercise bouts. While carbohydrate oxidation can arguably provide a superior substrate for exercise performance (i) due to its hypothetical improvements in energy efficiency of carbohydrate per unit of O_2_ consumed and (ii) its obligatory requirement to maintain exercise performance at very high intensities, we recently demonstrated (i) equivalent exercise performance despite distinct carbohydrate oxidation levels and (ii) maintenance of exercise performance at peak fat oxidation during exercise intensities > 85% VO_2max_, suggesting that substrate oxidation during exercise may not be a key determinant of exercise success [1,5].

Recently, we showed that carbohydrate-restricted high-fat diets are a feasible strategy to sustain 5 km running performance [13,14]. Moreover, we also documented that a low-carbohydrate, MCT-containing high-fat (LCHF) pre-exercise meal can elicit similar time to exhaustion during a simulated rucking march (TTE; ~1 h to volitional cessation) relative to isocaloric, high-carbohydrate low-fat (HCLF) nutrition [15]. The original rationale of the HCLF/LCHF pre-exercise nutrition study was to test energy-dense food items in a military-relevant setting to evaluate the utility of portable nutrition for long activities that require optimizing carry load and space to support task-relevant energy expenditure. While controlling for diet (i.e., mixed diet), our most recent work suggests that neither glycogen nor carbohydrate oxidation determines prolonged strenuous exercise bout performance and that minimal carbohydrate dosing (10 g/h) improves exercise performance through mitigating EIH [1].

Based on our prior findings, we were motivated to further probe whether substrate oxidation dictates exercise performance. Two pre-nutrition fueling methods, HCLF and LCHF, designed to elicit distinct CHO and FAT oxidation levels, were tested using TT aerobic events lasting <60 min. We thus conducted a series of studies—referred herein as *Study 1* and *Study 2*—to explore whether acute ingestion of LCHF or HCLF pre-exercise nutrition before a 5 km TT (*Study 1*) and a 10 km TT (*Study 2*) can modulate TT performance and intra-race exercise metabolism.

## 2. Methods

### 2.1. Experimental Design

**Figure 1:**
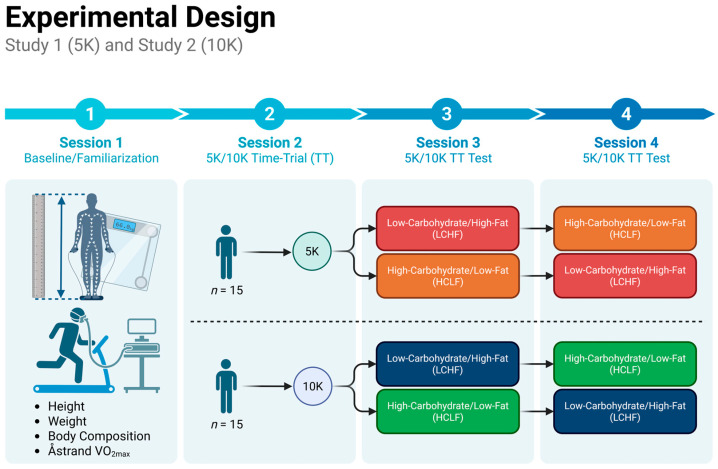
Experimental design.

A randomized, single-blind, counterbalanced study was used to assess the effects of LCHF vs. HCLC pre-exercise nutrition on performance during a 5 km TT (*Study 1*) and 10 km TT (*Study 2*) in 30 healthy, experienced recreational runners (Figure 1). The participants completed four sessions. Session 1 included familiarization with standard height, weight, and body composition measures, followed by a graded treadmill exercise test (Ȧstrand VO_2max_ test). Session 2 included performance testing of a baseline TT (without bar consumption). During sessions 3 and 4, the participants consumed either an LCHF or HCLF meal following an overnight fast. Three hours post-LCHF or HCLF meal ingestion, the athletes performed a TT. Blood metabolites (blood lactate, blood glucose, and blood ketones) were assessed prior to ingestion, 30 min post-ingestion (+30 min), 3 h post-ingestion, and immediately post-exercise. Perceptual measures for the rating of perceived effort (RPE) and affect were assessed throughout each TT. HR and respiratory gas exchange were monitored continuously during the graded exercise test and each TT. Before implementation, all procedures received IRB approval (117-2022/124-2023), and all subjects signed an informed consent document. The investigations were conducted in accordance with the principles outlined in the Declaration of Helsinki (1975, revised in 2013). All testing was conducted in the Grove City College Exercise Science Department Human Performance Laboratory.

Thirty recreational runners were allocated to participate in *Study 1* (*n* = 15) or *Study 2* (*n =* 15). Baseline anthropometry—weight, height, and body composition (electrical impedance)—and VO_2max_ was measured during session 1 prior to the experimental visits. Session 2 was designed to allow subjects to practice the 5 km or 10 km time trial (TT) to further familiarize them with the study protocol. Sessions 3 and 4 consisted of two experimental visits, where each subject consumed a low-carbohydrate high-fat (LCHF) and high-carbohydrate low-fat (HCLF) pre-exercise meal, in cross-over fashion, prior to their designated TT bout. There was a 1 week washout between sessions 3 and 4.

### 2.2. Participants

Two independent cohorts (*n* = 15/15) were recruited and assigned to *Studies 1* and *2* (Table 1). Participants were recruited directly from local running clubs and through community advertisements. Inclusion criteria stipulated the following: (1) completed a 5 km run in under 28 min (*Study 1*) and a 10 km run in under 55 min (*Study 2*) within the last three months, (2) running between 16 and 48 km per week, (3) aged between 18 and 35 years old, (4) had >2 years of running experience, (5) VO_2max_ ≥ 40 mL/kg/min, and (6) consuming a Standard American Diet [16]. Participants were excluded from our study if they had (1) a history of smoking, (2) any known metabolic, respiratory, or cardiovascular diseases, including diabetes, and (3) a presence of orthopedic, musculoskeletal, neurological, psychiatric disorders and/or any medical conditions that prevent exercise. The participants were instructed to refrain from exercise 24 h prior to a session, caffeine and alcohol consumption 48 h prior to a session, and food/drink for 3 h before each training session. Before enrolling in the investigation, participants were fully informed of any risks and discomforts associated with the experiments prior to giving their written informed consent to participate.

### 2.3. Pretrial Preparation

The participants were instructed to maintain their usual training frequency during each study intervention without increasing or decreasing the training load. The participants were instructed to maintain a training log (mode, duration, and intensity of each workout) for 1 week before the first experimental trial. They were provided with a copy of their pre-trial log and instructed to replicate the training routine during the intervention period. In addition, the participants were asked to record their training every week during the study (mode, duration, and intensity of each workout). To quantify training intensity, the participants were asked to record their session RPE (sRPE) after every training session [17] (pre-trial and within trial) using the OMNI Walk/Run 0–10 Perceived Exertion Scale [18]. The training load for each session was calculated by sRPE x duration of session (minutes) [19]. The sum of each session’s training load was used to quantify the weekly training load. The training load was assessed each week to measure compliance (Supplemental Table S1).

The participants’ habitual pre-trial and within-trial dietary intake was assessed weekly via integrative mobile technology (MyFitnessPal, San Francisco, CA, USA). The use of a mobile app for quantifying food intake has been previously shown to be an effective monitoring tool [20]. The subjects were asked to keep a detailed diary for 7 days prior to the start of the first experimental trial. The participants were provided with a copy of their pre-trial log and instructed to have the same dietary intake during the remainder of the study (weeks 1 and 2). During the familiarization session, the participants were given precise oral and written instructions individually on how to accurately record amounts and types of food and beverages (Supplemental Table S2).

### 2.4. Familiarization, Anthropometric Measurements, and Maximal Aerobic Capacity

The participants underwent an orientation involving TT practice and familiarization with the various measurement instruments, equipment, affect measures, and perceived exertion. Affect was measured using a validated 11-point Feeling Scale [21], with the participants informed that their responses should reflect the affective or emotional components of the exercise and not the physical sensation of effort or strain. The OMNI Walk/Run Perceived Exertion Scale [18] was used to measure the physical perceptions of exertion for the overall body (RPE-O). Following the orientation session, anthropometric measures were obtained, including height (cm), weight (kg), fat free mass (kg), and fat mass (% and kg). Height (cm) was measured using a physician’s scale (Detecto, Webb City, MO, USA). The participants’ body mass (kg) and body composition (fat and lean mass) were measured using a Tanita bioelectrical impedance analyzer (BIA) (MC-980Uplus, Tanita Corporation of America, Arlington Heights, TL, USA). Finally, the participants performed a graded exercise test to exhaustion on a motorized treadmill (Trackmaster TMX425C treadmill, Newton, KS, USA). Oxygen consumption (VO_2_) and carbon dioxide production (VCO_2_) were measured using an automated metabolic analyzer system (TrueOne 2400, ParvoMedics, Sandy, UT, USA) calibrated prior to each exercise test using standard calibration gases (16% O_2_ and 4% CO_2_). The participants wore a Polar heart rate monitor (H10, Polar Electro, Kempele, Finland) during exercise to measure their heart rate. After a thorough explanation of the experimental procedures, each participant was instructed to walk on the treadmill for 3 min as a warm-up at a self-selected speed (0% grade). Immediately following the 3 min warm-up, the speed was increased to 5–8 mph for 3 min (0% grade) to achieve the participants’ comfortable running pace. After 3 min of running at 0% grade, the grade was increased by 2.5% every 2 min throughout the test protocol, while speed was kept constant. The treadmill test was terminated by the subject at the point of volitional exhaustion. At the end of the test, the highest average VO_2_ value recorded over a 30 s period of exercise was considered the participant’s VO_2max_. A week after the familiarization visit, subjects returned to the laboratory to complete a baseline (i.e., practice) TT on the treadmill entailing no energy bar consumption.

### 2.5. Experimental Protocol

During the next two visits, the participants reported to the lab after an 8 h overnight fast to consume either an isocaloric low-carbohydrate high-fat (Keto Brick Inc., Bryant, AR, USA) or high-carbohydrate low-fat (2.4 services; ER 2400 Calorie Emergency Food Bar, Saint Louis, MO, USA) pre-exercise meal. The participants were randomly assigned to ingest ~1000 kcal of either LCHF or HCLF meal three hours before the performance test. The subjects were provided with 500 mL of water to consume ad libitum. Each condition was separated by at least one week. These pre-exercise nutritional meal options were chosen for having similar energy density, flavor profiles, and appearance, but distinct macronutrient profiles, which we previously demonstrated to produce distinct substate oxidation profiles [15]. A complete list of the macro-/micronutrients and their relative amounts is described in the supplement (Supplemental Table S3). Testing was single blinded; that is, the participants did not know the type of nutrition bar consumed. Blinding was achieved by removing the wrappers from the bars and dividing each bar into smaller bite-sized fragments. The LCHF and HCLF pre-exercise meals were similar in appearance (i.e., same color and consistency). We acknowledge that physiological cues such as differences in satiety could have provided indirect hints of meal type, but perceptual responses (RPE and affect) were monitored throughout to evaluate any expectancy influence.

### 2.6. Blood Sampling

Capillary blood samples for R-beta-hydroxybutyrate (*R*-βHB; Precision Xtra, Abbott Diabetes Care Inc., Almeda, CA, USA), blood glucose (Precision Xtra, Abbott Diabetes Care Inc., Almeda, CA, USA), and lactate concentrations (Lactate Plus, Nova Biomedical) were measured at baseline, 30 min post-meal ingestion (+30 min), 3 h post-meal ingestion (immediately before start of TT), and immediately following the TT. Samples were collected using a lancet following the cleaning of the fingertip with an alcohol swab and then dried. The first droplet was wiped away with a cotton swab to remove any alcohol, and the subsequent droplets were used for analysis.

### 2.7. 5 Km and 10 Km Running Time Trials

To determine exercise performance, the participants performed a 5 km (*Study 1*) and 10 km (*Study 2*) running TT on a motorized treadmill (TMX425C treadmill; Trackmaster, Newton, KS, USA). Before the start of the run, the participants completed a 5 min self-paced warm-up run. The participants were instructed to finish the run as fast as possible. The gradient was set at 0.0% grade. The participants were provided with feedback on the distance [at regular 500 m intervals (*Study 1*) and at regular 1000 m intervals (*Study 2*)] covered during each TT and were not informed of the overall performance time until completion of the study. During the TT, the participants were permitted to volitionally adjust their speed during the TT via control buttons located on the treadmill. The speed indicator and timing devices were concealed from the participants’ view throughout the TT; thus, treadmill pace was adjusted by the participants depending on perceived exertion and subjective feelings [22]. Heart rate (Polar Electro, Kempele, Finland) was recorded at 500 m (*Study 1*) and 1000 m (*Study 2*) intervals during the TT. Metabolic gases were continuously collected during the entire TT using a metabolic cart for the assessment of RER, VO_2_, VCO_2_, VE, VT, RR, and substrate oxidation.

### 2.8. Perceptual Measurements

The participants’ RPE (RPE-Overall) and affect (Feeling Scale) were recorded at 500 m (*Study 1*) and 1000 m (*Study 2*) intervals during the TT protocol. Ratings of perceived exertion and affect for the entire exercise session (session RPE and session affect) were obtained 5 min following the test. Thirst and gut fullness levels were measured using a previously validated instrument [23] at baseline, 30 min post-bar ingestion, 3 h post-energy bar ingestion and post-TT. Responses were recorded on a scale of 1–7 (1: ‘not thirsty at all’, 7: ‘very, very thirsty’) and 0–10 (0: ‘empty’, 10: ‘extremely full’), respectively.

### 2.9. Statistical Analysis

Statistical analyses were performed using SPSS version 28.0 (SPSS Inc., Chicago, IL, USA). Statistical significance was set a priori at *p*  <  0.05. Descriptive statistics were calculated for all variables. Normality and absence outliers were verified using the Shapiro–Wilk test, normality plots, and residual plots. Performance, physiological, and perceptual data collected during the TT (running time, mean exercise heart rate, RER, VO_2_, VCO_2_, V_E_, RR, carbohydrate and fat oxidation rates, affect, RPE-Overall, session RPE, and session affect) were analyzed using a paired-samples Student’s t-test as overall means throughout the exercise session. A 2 (condition: LCHF vs. HCLF)  ×  4 (time: baseline, 30 min post-ingestion, 3 h post-ingestion, immediately post-exercise) repeated measures ANOVA was conducted to assess the effect of time, treatment, and interaction between time and treatment on capillary glucose, lactate, ketones, and subjective measures of thirst and gut fulness; the mean results are summarized in the supplemental tables (Supplemental Tables S4 and S5). A one-way repeated measures ANOVA was used to analyze differences over time for training load during the intervention. A paired-samples Student’s t-test was used to analyze nutrient intake during the intervention. Post hoc analyses of significant main and interaction effects were conducted where appropriate using the Bonferroni adjustment to determine which conditions were significantly different. The assumption of sphericity was confirmed using Mauchly’s test. Greenhouse–Geisser epsilon corrections were used when the sphericity assumption was violated. Effect sizes (Cohen’s *d*) were calculated and interpreted as small effect > 0.2; medium effect > 0.5; large effect > 0.8 All data are reported as mean  ±  SD.

## 3. Results

### 3.1. Performance & Metabolic Responses

**Figure 2:**
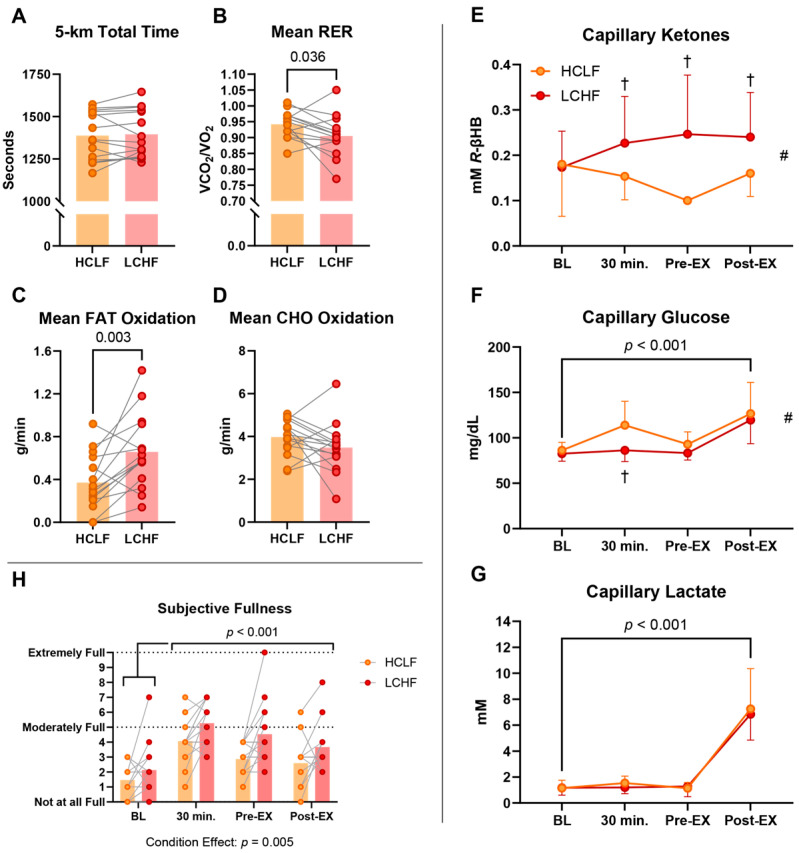
*Study 1* results. ^†^ = significant difference between LCHF and HCLF at the indicated timepoint. ^#^ = significant main effect of condition.

**Figure 3:**
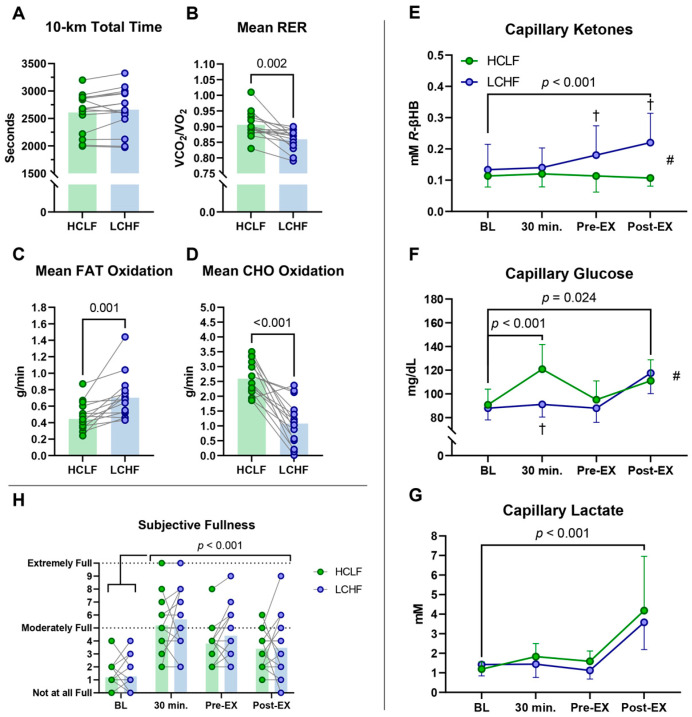
*Study 2* results. ^†^ = significant difference between LCHF and HCLF at the indicated timepoint. ^#^ = significant main effect of condition.

All subjects were included in the final analysis (*Study 1*; *n* = 15/*Study 2*; *n* = 15). The results are summarized in the table (Table 2) and figures below (Figure 2 and Figure 3). Despite distinct substrate oxidation profiles, there were no differences in either 5 km (*Study 1*; Figure 2A) or 10 km (*Study 2*; Figure 3A) TT performances. Although the TT times in *Study 2* showed a non-significant difference (*p* = 0.118), this falls well above the threshold for statistical significance and should be interpreted cautiously. Overall, despite distinct substrate oxidation profiles, TT performance did not differ between conditions. The LCHF pre-exercise meal elicited 4% lower RER values (*p* = 0.037) and 77% higher mean fat oxidation rates (*p* = 0.003) during the 5 km TT in *Study 1*, whereas CHO oxidation was comparable between conditions (Figure 2B–D). Congruent with *Study 1*, the LCHF meal also elicited 5% lower RER values (*p* = 0.002) (Figure 3B), 58% greater mean fat oxidation rates (*p* < 0.001), and 68% lower carbohydrate oxidation rates (*p* < 0.001) (Figure 3C,D) relative to the HCLF meal during *Study 2*. There were no significant differences between conditions for HR, VO_2_, VT, and perceptual session affect.

There were no differences between subjects at baseline for *R-*βHB. In *Study 1*, there was no main effect of time for *R-*βHB (Figure 2E). The significant condition effect revealed that the LCHF meal promoted approximately two-fold greater mean circulating *R-*βHB concentrations relative to the HCLF meal (*p* < 0.001). The significant interaction effect detected significant post hoc differences between the LCHF and HCLF meals 30 min post-ingestion, pre-exercise, and post-exercise. In *Study 2*, the significant main effect of time revealed similar *R-*βHB kinetics from baseline to 30 min post-ingestion and thereafter (Figure 3E). The condition effect and interaction were congruent with *Study 1*, where the LCHF meal increased mean *R-*βHB beyond the HCLF meal, whereas the post hoc tests indicated significant differences between conditions at pre- and post-exercise timepoints (all *p* < 0.05).

### 3.2. Glucose

There were no differences between the subjects at baseline for blood glucose. The significant main effects and interactions in both studies revealed that the LCHF meal did not influence blood glucose at rest, whereas the HCLF meal ingestion increased blood glucose ~30% relative to baseline, specifically in *Study 2* (*p* < 0.001) (Figure 2F and Figure 3F). When examined by condition across both studies, the HCLF meal elicited 9–13% (8–12 mg/dL) greater mean glucose responses compared to the LCHF meal (*p* < 0.01). Exercise predictably increased blood glucose significantly beyond resting values, independent of the supplement consumed prior to exercise.

### 3.3. Lactate

There were no differences between the subjects at baseline for blood lactate. Neither bar influenced circulating blood lactate levels post-ingestion or at rest. The TT test in both studies predictably increased blood lactate from pre- to post-exercise, independent of the assigned condition (Figure 2G and Figure 3G).

### 3.4. Perceptual Responses

During *Study 1*, the main effect of time for thirst reflected an increase in thirst sensation (BL vs. Post-EX: 28%; *p* < 0.001) depending on exercise termination and independent of condition. On the other hand, the LCHF meal was perceived as ~30% more filling than the HCLF meal (*p* = 0.005) (Figure 2H). The effects of thirst and hunger were congruent with *Study 2*, whereby thirst increased depending on exercise and independent of condition, with a significant post hoc difference pre-exercise, indicating a lower propensity for thirst in the LCHF meal condition (LCHF vs. HCLF: 3.3 ± 1.2 vs. 4.1 ± 0.8; −20%; *p* = 0.004) (Figure 3H). The participants perceived both bars as equally filling in *Study 2*.

## 4. Discussion

The purpose of this investigation was to determine whether substrate oxidation was a key determinant of middle-distance running performance. While controlling diet, activity, and body weight, including glycogen and EIH confounders, we fed the participants either an LCHF or HCLF in crossover fashion. As designed, the LCHF meal elicited greater fat oxidation during both 5 km and 10 km TTs. These substrate oxidation profiles during exercise were further confirmed by the lower RER in the LCHF meal. LCHF meal also increased capillary *R-*βHB relative to HCLF meal in both studies. Despite distinct substrate oxidation and metabolite profiles, we revealed no differences in TT performance, suggesting that substrate oxidation did not dictate running performance, in line with recent findings that tested whether substrate oxidation is a key determinant of exercise performance [1].

We utilized a fed-state design, coupled with daily food logs to ensure adherence, for subjects who habitually consumed high-carbohydrate diets. This design was intended to mitigate any differences in liver or muscle glycogen prior to exercise performance testing. We used a 5 km and 10 km middle-distance TT design as their corresponding time course and intensity would not allow for the confounding initiation of EIH, a key determinant of exercise performance [1,3], and implemented two distinct pre-exercise nutritional meals with similar calories, flavor, and appearance, but with previously demonstrated ability to produce distinct substrate oxidation profiles to closely examine the preferential substrate oxidation shift on exercise performance [15]. While *Study 2* produced a non-significant *p*-value (*p* = 0.118), this should not be interpreted as evidence of a performance benefit. The totality of the results across both studies supports the primary conclusion that isoenergetic HCLF and LCHF meals yield distinct metabolic responses without affecting middle-distance running performance.

**Figure 4:**
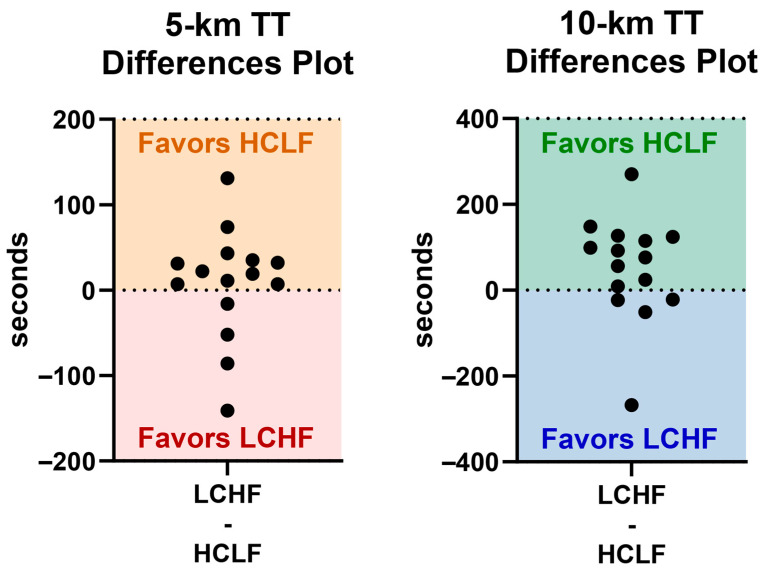
Individual responses to HCLF/LCHF meals on TT performance.

The TT results must be viewed with the understanding that all participants—in both *Study 1* and *Study 2*—consumed isonitrogenous habitual mixed diets, comprising primarily carbohydrates, prior to enrolling in the study. As shown in Figure 4, the addition of a standardized HCLF pre-race meal produced an additional benefit over an LCHF meal for most participants in both studies. The individual responses suggested that approximately 73% of participants ran faster 5 km/10 km TT races compared to the 27% who responded better on the LCHF meal. Our results are corroborated by others, who similarly did not find differences between acute pre-race high- and low-carbohydrate manipulations [4]. It is important to mention that acute vs. chronic (>6 weeks) diet implementation has a more profound effect on sport physiology, evidenced by glycemia and ketonemia normalization [1,13], peak fat oxidation capacity (~1.6 g/min; ~860 kcal/h) [24], and preferential fuel-substrate shifts for long-distance events [12]. Within the acute context presented herein, it is important to examine the results at the individual level to assist coaches and sport dieticians in developing personalized/precision-nutrition strategies for athletes to (1) augment high-responder performance and (2) identify other nutritional strategies for non-responders.

The significant difference in carbohydrate content between meals (Δ: 115 g) elicited a steadier glucose response during the LCHF meal relative to the HCLF meal. Although we did not measure insulin directly, we speculate, based on substrate oxidation differences and capillary blood glucose kinetics, that the LCHF meal plausibly reduced the insulinogenic response to the food challenge, thereby preserving greater fat oxidation rates. Compared to the prior literature looking at TT performance after low-glycemic index products were consumed prior to running [25,26,27], our results from this study fall in line with the neutral effects (i.e., no unique advantage to either strategy) reported in prior systematic and meta-analytical evidence [28]. However, congruent with our 70–80% VO_2max_ aerobic TT findings, low carbohydrate availability does not affect substrate oxidation rates negatively [10]. These results must, however, be framed within an acute feeding context. Chronic (i.e., months) adherence to low-carbohydrate nutrition can shift peak fat oxidation robustly to the right—from 50–60% VO_2max_ to 80–90% VO_2max_ [12]—owing to a basally elevated fat oxidation rate and propensity to recruit lipids towards energy expenditure [24,29]. Collectively, these lines of evidence demonstrate that (1) carbohydrates may not be an obligatory fuel for exercise intensities > 65% VO_2max_ and (2) low-carbohydrate pre-race nutrition confers greater metabolic flexibility during aerobic events without detrimental TT performance losses.

The 20 g of MCT contained within the LCHF meal modestly increased the synthesis of ketones into a range below nutritional ketosis (>0.5 mM capillary *R*-βHB). A 25–30 g MCT dose has reliably been shown to elevate *R*-βHB into a 0.5–1.0 mM range [30,31], which in part aligns with the ~0.3 mM *R*-βHB peak detected during *Studies 1* and *2*. Although this detected increase meets the original hypothesis expectations that an LCHF meal would promote *R*-βHB beyond an HCLF meal, this relatively low level of ketosis change was not associated with TT performance. Whereas prior research has shown improvements in cycling time trial performance bouts with MCT inclusion [32], contrasting evidence suggests that MCT has no effect or may elicit ergolytic TT performance effects via gastrointestinal distress [33,34]. Based on prior literature, incorporating MCTs into the LCHF meal allowed for acute manipulation of metabolite profiles while mitigating any direct effect of MCT on TT performance.

The fact that the subjects perceived the LCHF meal as more filling than the HCLF meal during *Study 1* was a surprising exploratory outcome. First, the subjects ingested 70% more HCLF meal product by mass relative to the LCHF meal prior to exercise (HCLF meal–LCHF meal: 57 g). Secondly, studies indicate that carbohydrates tend to have a stronger effect on satiety than lipids, whereby high-carbohydrate preloads have been shown to suppress subsequent food intake more effectively than high-fat preloads, revealing a relative insensitivity to the satiety value of fat [35,36]. With respect to running, increased carbohydrate and protein intake has been linked to the successful completion of ultra-endurance events by reducing the fractional utilization of maximal oxygen uptake and satiating hunger [37]. Collectively, a lower sensation of fullness after the HCLF meal preload should have (1) increased the sensation of fullness beyond the LCHF meal and (2) improved TT performance owing to improved hunger control. It is possible that the overall LCHF meal composition, although predominantly lipid-based, influenced hunger responses not solely through total fat content but also through its co-ingestion with protein [38] and, to a lesser degree, the presence of *R*-βHB [39]. Considering that TT performance was matched between conditions, it is unclear whether increased fullness conferred by the LCHF meal resulted in an adaptive response that can be transferred to longer running events.

Since the participants enrolled in *Studies 1* and *2* originated from two different cohorts, the between-study comparisons must be viewed with the understanding of additional between-subject variability. Secondly, we enrolled male cohorts for both studies, and whether these findings can extend to females requires further probing. Lastly, although the meals were provided in blinded form, physiological cues such as differences in satiety could have partially revealed the condition. Importantly, RPE and affect did not differ between meals (Table 2), suggesting minimal expectancy influence on performance. Several strengths to minimize potential variability included matching the *Study 1* subjects’ age, physical characteristics, VO_2max_, a priori diet (macronutrient and total energy), and training experience before enrolling subjects in *Study 2*.

## 5. Conclusions

Our RCT crossover design leveraged an LCHF pre-exercise meal, which significantly increased fat oxidation and lowered the respiratory exchange ratio compared to an HCLF meal while controlling for confounders such as glycogen depletion and exercise-induced hypoglycemia, utilizing a fed-state middle-distance exercise performance TT. Despite distinct substrate oxidation and metabolite profiles, our findings demonstrate no difference in exercise performance, challenging the notion that substrate oxidation is a key determinant of exercise performance. Future research should explore individual variability and alternative precision-nutrition strategies to better understand the multifactorial determinants of endurance performance.

## Figures and Tables

**Table 1:** Participant descriptives.

Characteristics	*Study 1* (5 km TT) *N* = 15	*Study 2* (10 km TT) *N* = 15
Age (years)	20.8 ± 3.5	31.2 ± 10.9
Height (cm)	182.0 ± 10.1	176.4 ± 6.4
Bodyweight (kg)	81.3 ± 12.4	78.6 ± 7.8
Body Fat (%)	14.0 ± 4.6	13.4 ± 4.4
Fat Free Mass (kg)	69.3 ± 8.7	73.4 ± 23.4
Fat Mass (kg)	11.7 ± 5.0	12.8 ± 8.1
BMI (kg/m^2^)	24.6 ± 3.5	25.4 ± 3.3
Mean exercise/week (min)	377.3 ± 195.4	318.8 ± 152.1
Exercise experience (years)	10.5 ± 5.4	13.7 ± 11.6
VO_2max_ (mL/kg/min)	58.3 ± 6.2	54.51 ± 5.9
Familiarization TT (min)	24.6 ± 3.2	45.7 ± 5.6

Data shown as mean ± SD. BMI, body mass index; TT, time trial; VO_2max_, maximal oxygen consumption.

**Table 2:** Time trial (TT) results.

Variable	*Study 1* (5 km TT)				*Study 2* (10 km TT)			
	LCHF	HCLF	*p*-Value	Effect Size	LCHF	HCLF	*p*-Value	Effect Size
TT performance (min)	23.3 ± 2.3	23.1 ± 2.4	0.646	0.06	44.4 ± 6.6	43.5 ± 6.1	0.118	0.14
CHO oxidation (g·min^−1^)	3.49 ± 1.17	3.98 ± 0.84	0.169	0.38	1.06 ± 0.77	2.59 ± 0.56	**<0.001**	2.27
FAT oxidation (g·min^−1^)	0.68 ± 0.34	0.37 ± 0.26	**0.003**	0.72	0.70 ± 0.26	0.44 ± 0.22	**0.001**	1.08
RER	0.90 ± 0.06	0.94 ± 0.04	**0.037**	0.78	0.85 ± 0.03	0.91 ± 0.04	**0.002**	1.69
VO_2_ (L·min^−1^)	3.85 ± 0.42	3.69 ± 0.53	0.337	0.33	2.88 ± 0.45	2.83 ± 0.41	0.388	0.12
VO_2_ (mL·kg^−1^·min^−1^)	47.7 ± 5.7	46.2 ± 7.5	0.391	0.23	36.9 ± 7.2	36.5 ± 6.3	0.453	0.01
VCO_2_ (L·min^−1^)	3.48 ± 0.44	3.48 ± 0.50	0.984	0.00	2.36 ± 0.37	2.57 ± 0.36	**0.018**	0.58
V_E_ (L·min^−1^)	107.5 ± 12.5	102.8 ± 15.8	0.412	0.33	80.2 ± 16.7	79.5 ± 13.9	0.680	0.05
RR (breath/min)	49.7 ± 17.3	46.8 ± 11.7	0.446	0.20	41.6 ± 7.24	41.3 ± 6.21	0.740	0.04
V_**T**_ (L)	2.51 ± 0.47	2.43 ± 0.44	0.455	0.18	1.99 ± 0.34	1.91 ± 0.31	0.142	0.25
HR (b·min^−1^)	181.9 ± 8.1	180.1 ± 8.8	0.196	0.21	160.9 ± 11.8	157.5 ± 12.7	0.117	0.28
RPE-O	6.4 ± 1.4	6.2 ± 1.2	0.167	0.15	5.9 ± 1.4	6.0 ± 1.3	0.704	0.07
Affect	0.2 ± 1.8	0.4 ± 2.1	0.606	0.10	0.8 ± 1.1	1.1 ± 1.3	0.329	0.25
Session RPE	7.3 ± 1.4	7.3 ± 1.5	0.806	0.00	7.87 ± 1.8	7.87 ± 1.3	1.000	0.00
Session Affect	−0.5 ± 1.8	0.0 ± 2.5	0.477	0.23	1.0 ± 1.2	0.0 ± 2.3	0.264	0.54

Data shown as mean ± SD. TT = time trial; HR = heart rate; RPE-O = RPE for overall body; RPE = rating of perceived exertion (OMNI rating of exertion); RER = respiratory exchange ratio; RR = respiratory rate; V_T_ = tidal volume; effect sizes (Cohen’s d): <0.2 = trivial; 0.2 to 0.49 = small; 0.5 to 0.79 = moderate; >0.8 = large. Bold *p*-values denote significant differences (*p* < 0.05). *N* = 15, *Study 1*; *n* = 15, *Study 2*.

## Data Availability

The data that support the findings of this study are available from the corresponding author upon reasonable request.

## Supplementary Materials

The following supporting information can be downloaded at: https://www.mdpi.com/article/10.3390/nu17172771/s1, Table S1: Training Load; Table S2: Nutrient Intake; Table S3: Pre-Exercise Meal Characteristics; Table S4: Blood Metabolites; Table S5: Thirst and Fullness Measures.
