# Supplementary material for: Substrate Oxidation Does Not Influence Middle Distance Running Performance: A Randomized Controlled Crossover Trial
Source: Nutrients. 2025 Aug 27;17(17):2771. doi: 10.3390/nu17172771 (PMC12430111; doi:10.3390/nu17172771)
Supplement: Supplementary file 1 [file nutrients-17-02771-s001.zip › nutrients-3830347-supplementary.pdf]

Supplemental Tables S1. Training Load.

**Training Load (RPE x min)**	**Pre-Trial**	**Week 1**	**Week 2**	**One-way ANOVA (*p*-value)**
Study 1 (5-km TT)	2227 ± 992.4	2280 ± 1084.9	2151.9 ± 869.0	0.843
Study 2 (10-km TT)	2244 ± 1214	2077 ± 1253	2304 ± 1354	0.627

Data shown as mean ± SD.

Table S2. Nutrient Intake.

	Study 1 (5-km TT)			Study 2 (10-km TT)		
**Variable**	**Week 1**	**Week 2**	***p*-value**	**Week 1**	**Week 2**	***p*-value**
Energy (kcals·day^{-1})	2811.2 ± 706.7	2727.3 ± 617.6	0.517	2252.1 ± 686.0	2307.2 ± 738.9	0.471
Carbohydrate (g)	266.9 ± 103.3	259.7 ± 66.7	0.729	236.7 ± 55.3	235.9 ±68.0	0.946
Protein (g)	166.3 ± 61.3	156.6 ± 66.8	0.256	120.5 ± 44.2	119.8 ± 48.3	0.912
Fat (g)	120.8 ± 34.3	116.3 ± 34.5	0.651	98.8 ± 29.9	98.2 ± 28.4	0.921
Carbohydrate (%)	37.3 ± 8.3	38.9 ± 7.5	0.578	41.3 ± 7.5	40.0 ± 5.9	0.389
Protein (%)	23.7 ± 6.1	23.0 ± 6.1	0.557	21.3 ± 5.7	20.1 ± 5.2	0.461
Fat (%)	37.5 ± 8.6	39.1 ± 10.2	0.631	37.1 ± 7.9	37.4 ± 5.5	0.914

Participants (n = 15; Study 1; n = 15; Study 2) adhered to a Standard American Diet (Shan et al., 2019) through the intervention timeline. Data shown as mean ± SD.

Table S3. Pre-Exercise Meal Characteristics.

**Description**	**LCHF Meal**	**HCLF Meal**	**Net Difference (LCHF – HCLF)**
Total Energy (kcal)	1,000	1,000	0
Total Weight (g)	150	207	-57
Fat (g)	90	46	44
Saturated Fat (g)	62	22	40
Medium Chain Triglycerides (g)	20	0	20
Carbohydrates (g)	12	127	-115
Sugar (g)	2	39	-37
Fiber (g)	7	5	2
Protein (g)	30	17	13

Table S4. Blood Metabolites

Variable	Condition	Timepoint				*2x4 RM ANOVA*
		BL	+30min	Pre-EX	Post-EX	
Study 1 (5-km TT)						
R-βHB (mmol·L^{-1})	HFEB	0.17 ± 0.08	0.22 ± 0.10	0.25 ± 0.13	0.24 ± 0.09	Time, *P*=0.372<br>Condition, *P*=0.008<br>Interaction, *P*=0.003
	HCEB	0.18 ± 0.11	0.15 ± 0.05*	0.10 ± 0.00*	0.16 ± 0.05*	
Study 2 (10-km TT)						
R-βHB (mmol·L^{-1})	HFEB	0.13 ± 0.08	0.14 ± 0.06	0.18 ± 0.09	0.22 ± 0.09	Time, *P*<0.001<br>Condition, *P*=0.020<br>Interaction, *P<0.001*
	HCEB	0.11 ± 0.03	0.12 ± 0.04	0.11 ± 0.05*	0.11 ± 0.02*	
Study 1 (5-km TT)						
Glucose (mg·dL^{-1})	HFEB	82.5 ± 8.3	86.3 ± 12.4	83.3 ± 7.8	119.5 ± 25.9	Time, *P*<0.001<br>Condition, *P*=0.005<br>Interaction, *P*= 0.042
	HCEB	86.3 ± 8.8	113.9 ± 26.4*	92.9 ± 13.7*	126.6 ± 34.4	
Study 2 (10-km TT)						
Glucose (mg·dL^{-1})	HFEB	87.9 ± 9.9	91.1 ± 10.7	87.9 ± 12.1	117.5 ± 17.3	Time, *P<0.001*<br>Condition, *P*=0.007<br>Interaction, *P<0.001*
	HCEB	90.7 ± 13.2	120.8 ± 20.8*	95.0 ± 15.9	111.1 ± 17.7	
Study 1 (5-km TT)						
Lactate (mmol·L^{-1})	HFEB	1.17 ± 0.57	1.19 ± 0.48	1.28 ± 0.79	6.84 ± 1.99	Time, *P*<0.001<br>Condition, *P*=0.005<br>Interaction, *P*=0.019
	HCEB	1.15 ± 0.60	1.53 ± 0.53	1.13 ± 0.45	7.25 ± 3.09	
Study 2 (10-km TT)						
Lactate (mmol·L^{-1})	HFEB	1.42 ± 0.57	1.44 ± 0.68	1.12 ± 0.44	3.58 ± 1.39	Time, *P*<0.001<br>Condition, *P*=0.118<br>Interaction, *P*= 0.210
	HCEB	1.18 ± 0.38	1.83 ± 0.66	1.59 ± 0.52	4.18 ± 2.78	

Data shown as mean ± SD. Finger capillary blood glucose, *R*-β-hydroxybutyrate, and lactate were assessed across four timepoints (n = 15). BL = baseline. +30min = 30 min post ingestion. EX = exercise. HFEB = high-fat energy bar; HCEB = high-carbohydrate energy bars. * = p < 0.05 from HCEB at the indicated timepoint.

Table S5. Thirst and Fullness Measures.

Variable	Condition	Timepoint				*2x4 RM ANOVA*
		BL	+30min	Pre-EX	Post-EX	
		Study 1 (5-km TT)				
Thirst	HFEB	3.9 ± 1.1	3.9 ± 1.2	3.7 ± 0.9	5.1 ± 1.3	Time, *P*<0.001<br>Condition, *P*=0.079<br>Interaction, *P*=0.406
	HCEB	3.5 ± 0.9	3.5 ± 0.9	3.8 ± 0.7	4.5 ± 1.8	
		Study 2 (10-km TT)				
Thirst	HFEB	4.1 ± 0.9	3.9 ± 1.2	3.3 ± 1.2	4.8 ± 0.6	Time, *P*<0.001<br>Condition, *P*=0.296<br>Interaction, *P*=0.009
	HCEB	3.5 ± 1.1	4.1 ± 1.0	4.1 ± 0.8*	5.2 ± 1.0	
		Study 1 (5-km TT)				
Fullness	HFEB	2.1 ± 1.7	5.3 ± 1.4	4.5 ± 2.0	3.7 ± 1.5	Time, *P*<0.001<br>Condition, *P*=0.005<br>Interaction, *P*=0.270
	HCEB	1.5 ± 1.2*	4.1 ± 1.4*	2.9 ± 1.1*	2.6 ± 1.5*	
		Study 2 (10-km TT)				
Fullness	HFEB	1.3 ± 1.3	5.7 ± 2.3	4.4 ± 2.1	3.4 ± 2.4	Time, *P*<0.001<br>Condition, *P*=0.323<br>Interaction, *P*=0.788
	HCEB	1.2 ± 1.1	5.2 ± 2.1	3.8 ± 1.6	3.4 ± 1.4	

Data shown as mean ± SD. Thirst scale (1- "not thirsty at all, 7- "very, very thirsty"); Fullness Scale (0- "empty", 10- "extremely full"). BL = baseline; +30min = 30 min post ingestion; EX = exercise; KB = ketogenic bar; CB = carbohydrate bars. * = $p < 0.05$ from CB at the indicated timepoint.
